# Supplementary material for: Metabolic Profiling of Wheat Seedlings Under Oxygen Deficiency and Subsequent Reaeration Conditions
Source: Int J Mol Sci. 2025 Nov 30;26(23):11610. doi: 10.3390/ijms262311610 (PMC12692663; doi:10.3390/ijms262311610)
Supplement: Supplementary file 1 [file ijms-26-11610-s001.zip › Yemelyanov ea-IJMS_2025-supplementary figures.pdf]

International Journal of
Molecular Sciences

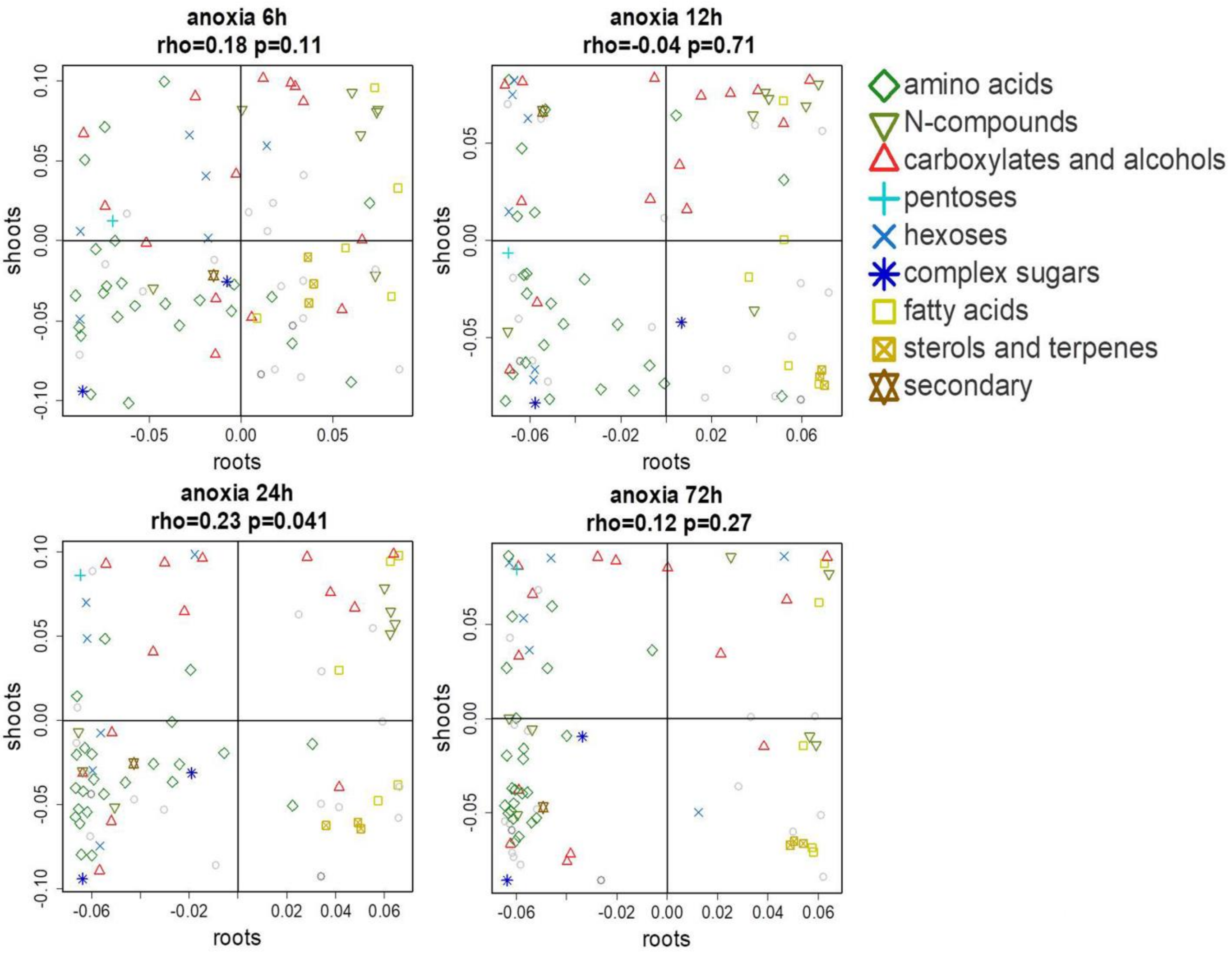

Figure S1. Comparison of roots and shoots metabolite dynamics in wheat seedlings under long-term anoxia (6-72 h). SUS (shared and unique structures) plot in the space of the loadings from OPLS-DA models.

International Journal of
Molecular Sciences

MDPI

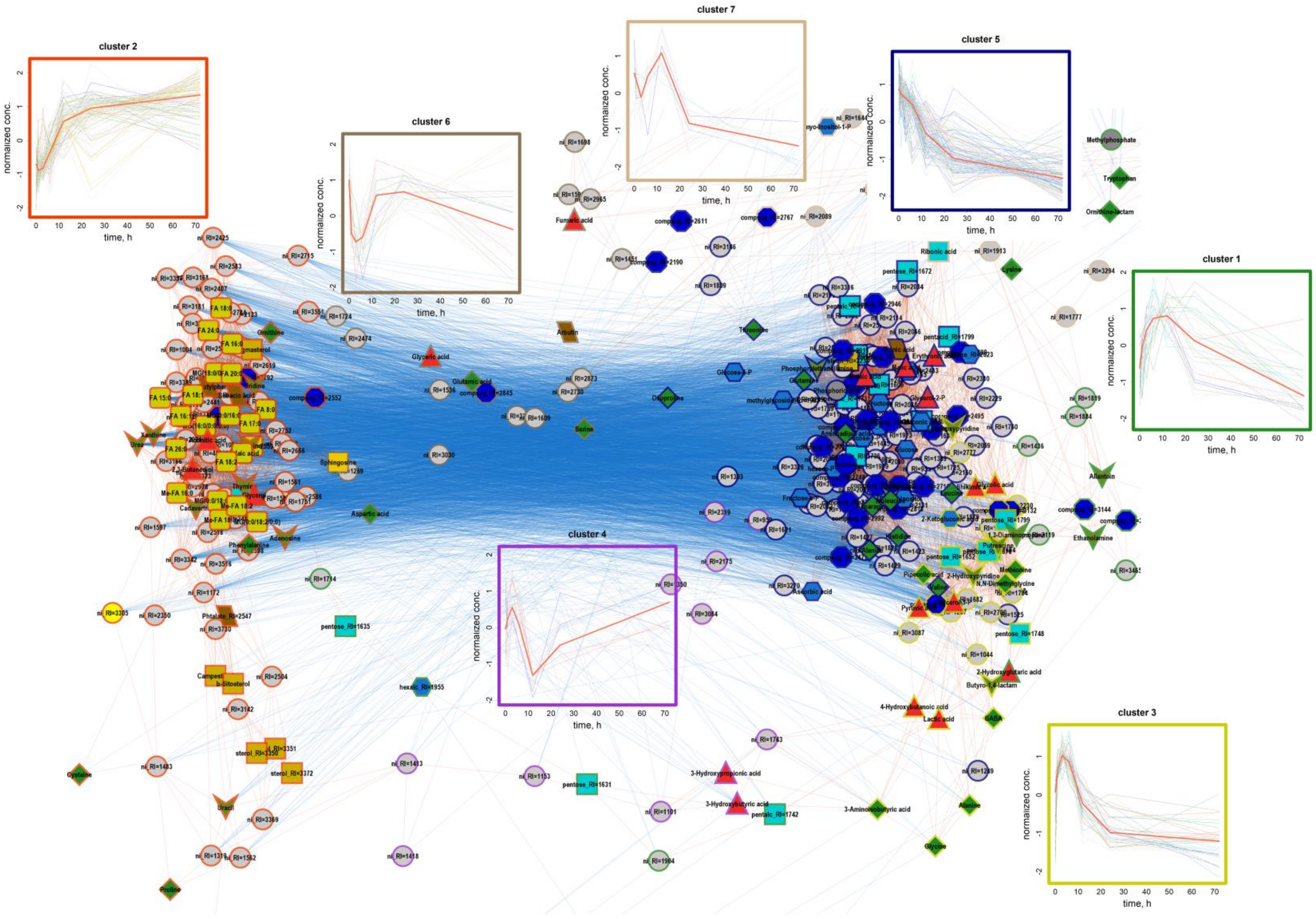

Figure S2. Patterns of metabolite dynamics in the roots of wheat seedlings during anoxia. Metabolites were mapped according to strong correlations (r>0.85). The edges denote correlations, blue – negative, red – positive. The dynamics patterns for the resulting clusters are illustrated in squares. Cluster membership is illustrated by the color outline of nodes and graphs.

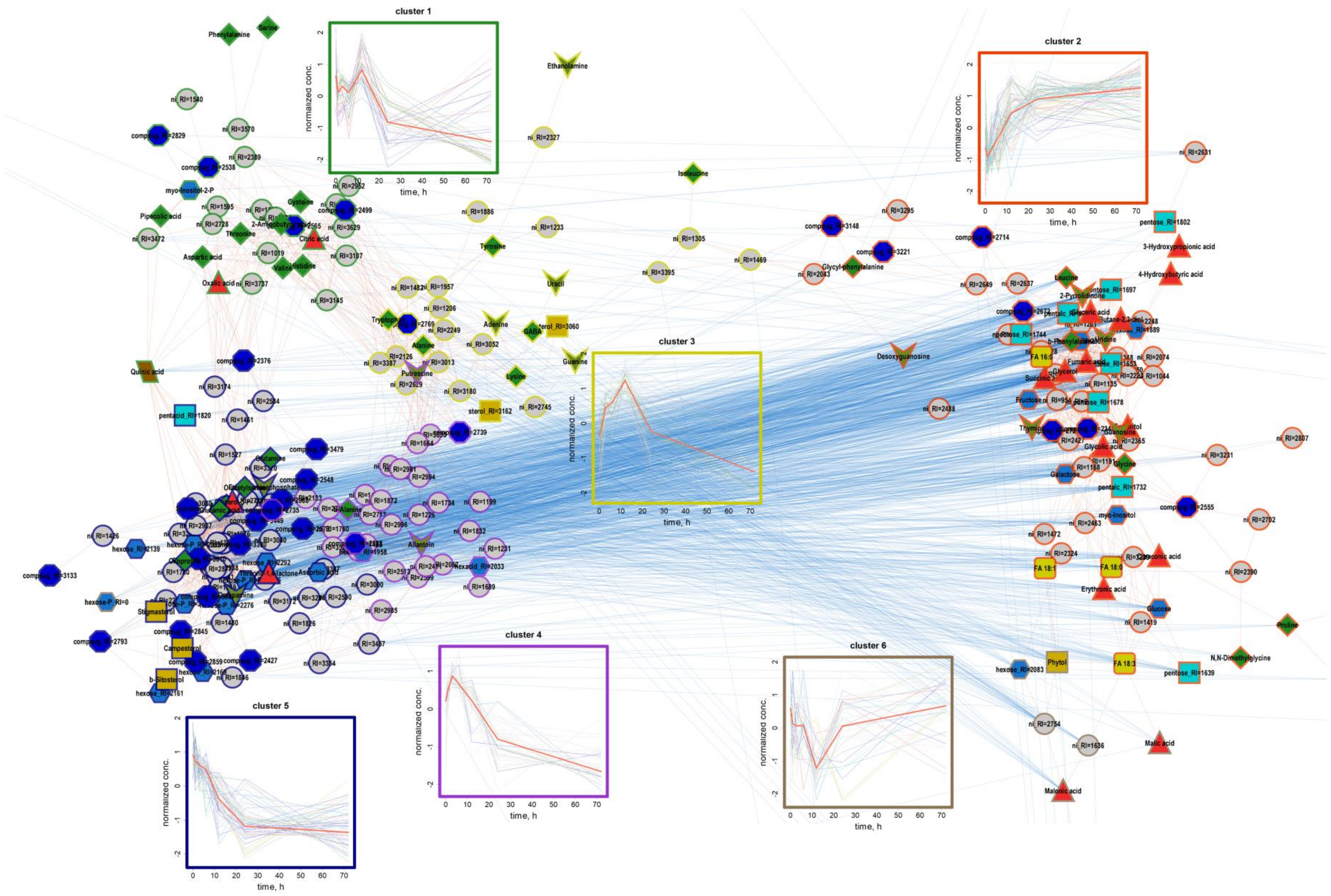

Figure S3. Patterns of metabolite dynamics in the shoots of wheat seedlings during anoxia. Metabolites were mapped according to strong correlations (r>0.85). The edges denote correlations, blue – negative, red – positive. The dynamics patterns for the resulting clusters are illustrated in squares. Cluster membership is illustrated by the color outline of nodes and graphs.

International Journal of
Molecular Sciences

MDPI

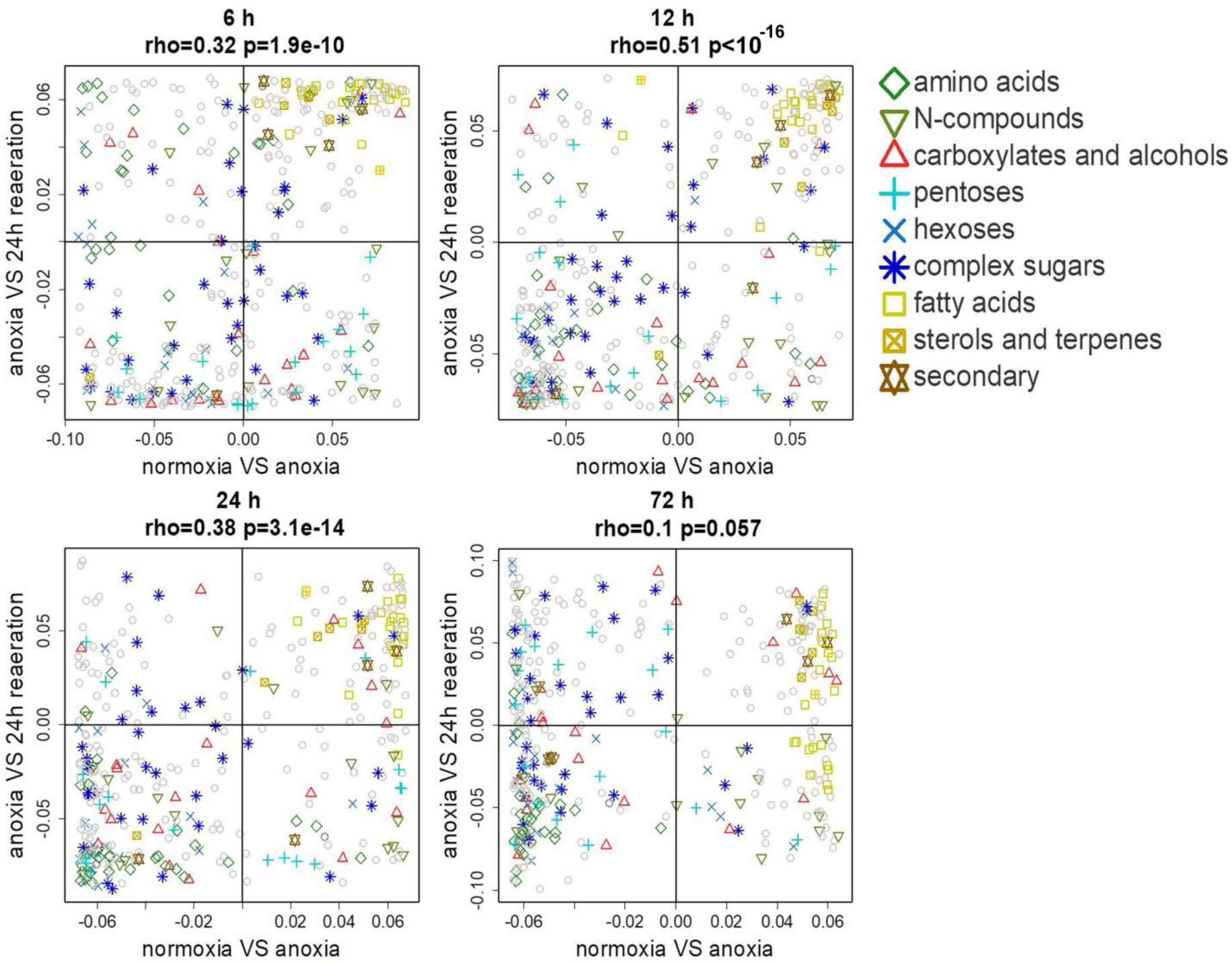

Figure S4. Comparison of the effects of anoxia and reoxygenation in the roots of wheat seedlings. SUS (shared and unique structures) plot in the space of the loadings from OPLS-DA models. Positive loadings correspond to a higher content under reaeration (when comparing effects of anoxia and reaeration) and anoxia (when comparing effects of normoxia and anoxia).

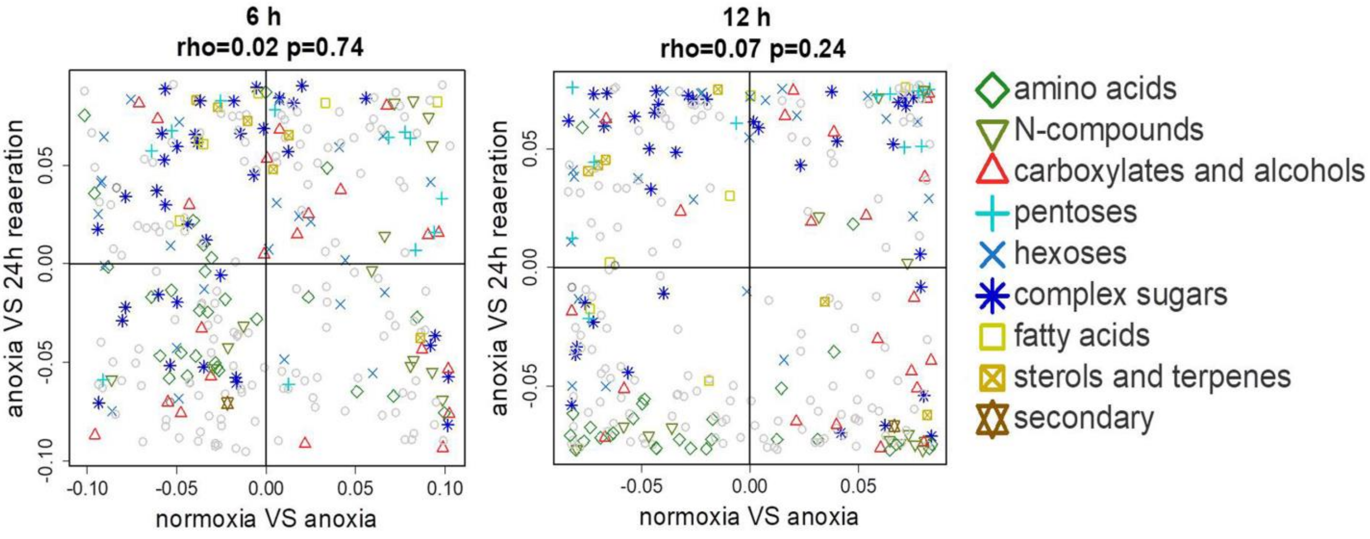

Figure S5. Comparison of the effects of anoxia and reoxygenation in the shoots of wheat seedlings. SUS (shared and unique structures) plot in the space of the loadings from OPLS-DA models. Positive loadings correspond to a higher content under reaeration (when comparing effects of anoxia and reaeration) and anoxia (when comparing effects of normoxia and anoxia).

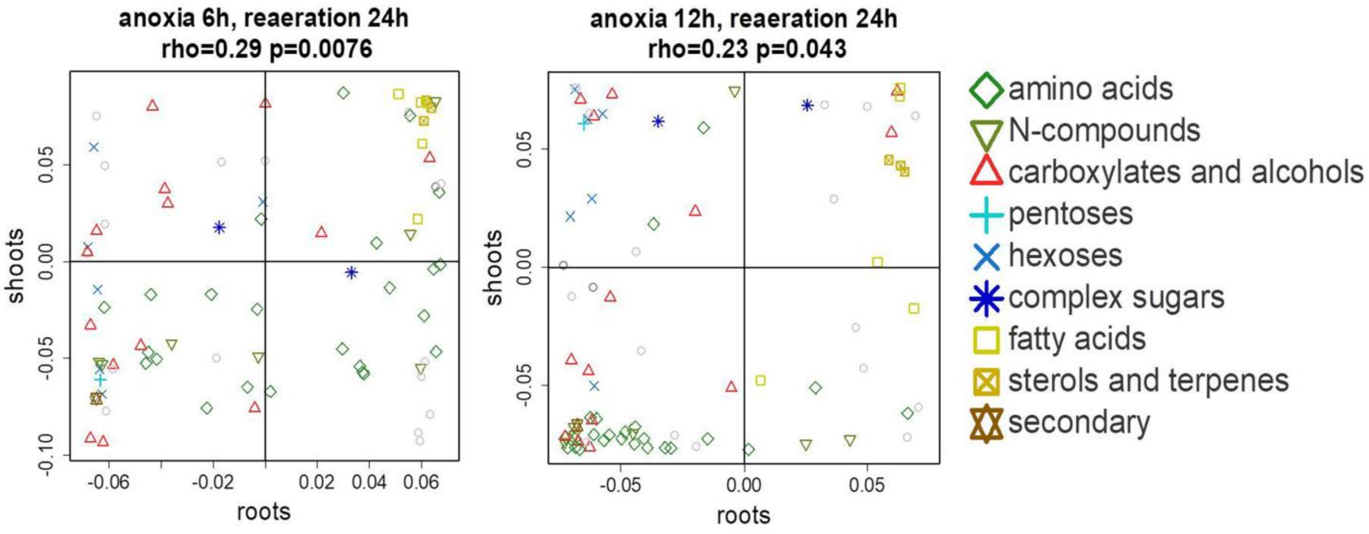

Figure S6. Comparison of the effects of reoxygenation on roots and shoots of wheat seedlings. SUS (shared and unique structures) plot in the space of the loadings from OPLS-DA models.
